# The pharmaceutical death-ride of dihydroartemisinin

**DOI:** 10.1186/1475-2875-9-212

**Published:** 2010-07-22

**Authors:** Frans Herwig Jansen

**Affiliations:** 1 Fondation ACT-ion Afrique. Brussels, Belgium

## Abstract

In the 2010 second edition of WHO's guidelines for the treatment of malaria, the relatively new fixed dose combination dihydroartemisinin-piperaquine is included as one of the recommended artemisinin combination therapies. However, experimental testing demonstrates that, due to its intrinsic chemical instability, dihydroartemisinin is not suitable to be used in pharmaceutical formulations. In addition, data show that the currently available dihydroartemisinin preparations fail to meet the internationally accepted stability requirements. At a time when many efforts aim to ban counterfeit and substandard drugs from the malaria market, the obvious question rises how WHO and public-private partnerships, such as Medicine for Malaria venture (MMV), can support the production and marketing of anti-malarial drugs that do not even meet the International Pharmacopoeia requirements?

## Background

In its recent 2010 edition of recommended malaria treatment, WHO recommends - among others - the use of a particular fixed dose artesunate-based combination therapy (ACT) composed of dihydroartemisinin (DHA) and piperaquine phosphate, known under the brand names Artekin^® ^or Duo-cotecxin^® ^(Holley-Cotec, China) [1]. The prefix "duo" is used because a single-ingredient preparation named Cotecxin, containing only DHA, had been previously manufactured and distributed for years in a massive manner all over Africa, strongly supported in official relief programmes by Chinese authorities. This product, as well as the newer Duo-cotecxin, requires closer attention. These are the only malaria treatments containing DHA instead of the more commonly used artemisinin-derivatives artesunate or artemether. Despite the fact that DHA is the most active derivative of artemisinin, this chemical entity is a product of major concerns from the ethical, regulatory and therapeutics points of view.

## Discussion

**Figure 1:**
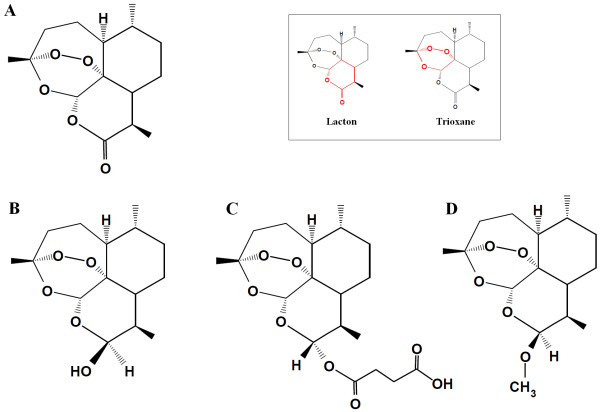
**Structure of artemisinin (A), dihydroartemisinin (B), artesunate (C) and artemether (D)**. The insert shows the lacton (for artemisinin) and trioxane functional groups (marked in red).

After the discovery in 1976 of the anti-malarial properties of artemisinin, extracted and crystallized from *Artemisia annua *leaves, it took several years before the structure of this substance (bruto molecular structure of C_15_H_22_0_5 _and mol. wt. 282.35) could be elucidated (Figure 1). X-ray crystallography indicated that this compound had a lactone structure [2]. This led immediately to the chemical reflex of making a lactol of this function by a selective reduction process. Such a lactol, given the trivial name dihydroartemisinin (DHA) offers interesting possibilities because it permits to substitute on the molecule. Esters and ethers are the obvious derivatives to be made and hence the structures of artesunate and of artemether were born. It was soon found that these substances exceeded artemisinin in intrinsic pharmacological action against malaria parasites. DHA received later the official generic name of artenimol. This chemical entity is obtained after e.g. a borohydride reduction of artemisinin in methanol at - 5°C. The reaction is stopped and the DHA is precipitated with water and acid. After washing and drying, a nearly 99% pure DHA is obtained. DHA behaves like an amorphous powder and has a melting point of 164°C.

**Figure 2:**
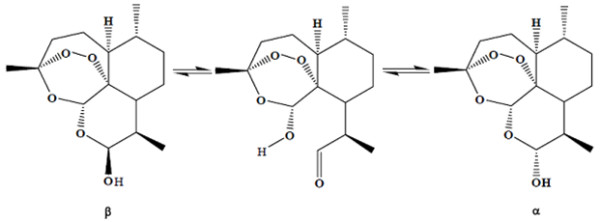
**Interconversion of α/β epimers of dihydroartemisinin with ring opening**.

Artemisinin and its derivative DHA is a fascinating composite. Artemisinin is synthesized by the plant *Artemisia annua*, and not less than seven of its carbon atoms are asymmetric, meaning that this molecule has unique stereochemical features. Strictly-speaking, it is a three cyclic ring system with a peroxide bridge build over a seven membered heterocyclic (oxygen containing) ring. In this ring, the peroxide function is sitting in a configuration called a tri-oxane. In addition, the molecule carries two other oxygens atoms forming a lacton function, and in DHA a lactol function. The stereochemistry of the molecule and each of its oxygen atoms play an important role in its action. The presence of a peroxide bridge on top of an oxygen containing seven membered ring is chemically quite remarkable, and these heterocyclic ring systems are known to be chemically rather unstable. In fact, this special ring system interacts with the lactol function of DHA. The lactol -OH group can exist in an alpha and beta position and they can switch from one position to another. This flip-flop is accompanied by ring opening of the lactol ring system (Figure 2) [3]. This phenomenon brings extra strain on the molecule, particularly on the seven membered ring carrying the peroxide function. When certain conditions are met, this leads to the destruction of the molecule. The total of processes involved in this breakdown is capable of creating a number of free radicals which can be formed biologically. A number of such free radicals with alkylating properties have been previously described, with some needing the presence of ferrous ions. The major and probably the most reactive free radical generated by DHA is singlet oxygen when released from the peroxide group. This singlet oxygen can exert all the specific and non-specific reactions linked to peroxides [4].

The chemical instability of DHA was subject of investigation by several groups. Haynes *et al *demonstrated that DHA is thermally labile posing a problem, not only in relation to product stability as defined in ICH (The International Conference on Harmonisation of Technical Requirements for Registration of Pharmaceuticals for Human Use) stability testing guidelines, but also because its special physicochemical properties and relatively poor oral bioavailability pose problems in its use in fixed pharmaceutical formulations [5]. In addition, it was demonstrated that DHA decomposes into several breakdown products of which two could be identified [6].

Experiments on the pure DHA raw material confirmed the instability of this molecule and led to the suspicion that this substance would have serious difficulties to survive pharmaceutical legislation when used in tablets. Pharmaceutical experiments with DHA showed that the making of a good tablet is not an easy task and several attempts were necessary for adequate formulation of DHA in an acceptable galenic form. When these problems were solved various types of tablets were made that could be manufactured industrially. The problem remained as to whether they would survive further stability testing and more particular if they would be of sufficient quality to survive tropical climate conditions. The critical questions were: do these tablets contain a minimum content of active pharmaceutical ingredient DHA at the end of shelf life? And practically, do these tablets in their original packaging, when kept at tropical conditions (30°C and 65-75% humidity), survive the shelf life period of two to three years? The current international pharmaceutical law is very strict and requires a minimum of 90% (and in some countries even 95%) API integrity throughout the shelf-life of the product. In addition, would breakdown products remain within the set limits of 2 per cent?

Initially, the tablets fulfilled all criteria of hardness, disintegration time, friability etc. However when the first samples of PVC/aluminium blistered tablets - kept under the conditions of stability testing - were submitted for analysis a few striking phenomena had occurred. Nearly all blisters showed black dot incrustations in the PVC (as if it were the result of a burning) and the tablets also showed black dot mottling. The tablets had been stored in stability rooms (30°C/65% relative humidity) and 40°C/75% relative humidity for accelerated stability testing. Upon analysis the content of DHA had fallen below 90% indicating that these tablets would never be suitable for a final production (Table 1).

**Table 1:** Outcome of stability studies performed on three batches of dihydroartemisinin tablets (Dynamax, Proxi Pharma, Belgium).

Time	Assay	Disintegration	Dissolution	Uniformity of mass	Average weight	Appearance
**Month**	**%**	**Minutes**	**%**			
	**(90-110)**	**(≤15)**				**(beige tablet)**

**Batch n° A - Accelerated stability studies: 40°C and 75% RH**						

**Zero**	97.4	conform	75	conform	0.2728	conform
**1**	99.3	conform	81	conform	0.2776	conform
**3**	**80.4**	conform	65	conform	0.2782	**not conform**

**Batch n° A - Real time stability studies: 30°C and 65% RH**						

**Zero**	97.4	conform	75	conform	0.2728	conform
**3**	95.6	conform	70	conform	0.2769	conform
**6**	**85.5**	conform	Not tested	conform	0.2786	conform

**Batch n° B - Accelerated stability studies: 40°C and 75% RH**						

**Zero**	91.7	conform	73	conform	0.2747	conform
**1**	93.9	conform	75	conform	0.2792	conform
**3**	**77.3**	conform	74	conform	0.2805	**not conform**

**Batch n° B - Real time stability studies: 30°C and 65% RH**						

**Zero**	91.7	conform	73	conform	0.2747	conform
**3**	100.9	conform	67	conform	0.2775	conform
**6**	**79.1**	conform	Not tested	conform	0.2803	conform

**Batch n° C - Accelerated stability studies: 40°C and 75% RH**						

**Zero**	93.7	conform	80	conform	0.2734	conform
**1**	95.0	conform	70	conform	0.2791	conform
**3**	**88.4**	conform	73	conform	0.2805	**not conform**

**Batch n° C - Real time stability studies: 30°C and 65% RH**						

**Zero**	93.7	conform	80	conform	0.2734	conform
**3**	91.2	conform	68	conform	0.2785	conform
**6**	**80.4**	conform	Not tested	conform	0.2806	conform

Assay performed by HPLC with reference standard; appearance assessed by macroscopic inspection; disintegration, uniformity of mass and friability determined according to European Pharmacopoea.

When DHA tablets were introduced on the African market, some manufacturers established a strong position in a number of countries and large quantities of these drugs were donated to the population. In order to evaluate the quality of these products, samples were purchased in several pharmacies in Africa (before 2006) and sent to the laboratory for quality testing. The samples were analysed using the HPLC method (related substances, method A) described in the Monograph Artenimol (International Pharmacopoiea, third edition, volume 5). The results of the analyses were astonishingly bad and none of the tablets were conform to the requirements of the International Pharmacopoeia. In all samples tested, the concentration of related substances (breakdown products) was above 2%, in some samples even above 20%. In many samples the content of DHA had fallen to values of about 50% of the initial API content. From a medical point of view, this has serious and negative consequences for the population at risk for malaria. DHA tablets contain ideally 60 mg of active ingredient and the dosing scheme in monotherapy is such that with this low dosage it is difficult to ascertain adequate therapy in an African population (as compared to e.g. tablets containing 100 mg of Artesunate). In general, the recommended dose for DHA tablets should be significantly higher. If, however, the content of the active ingredient in these tablets significantly drops, the therapeutic potential of such tablets can be seriously questioned. Even more, it could be argued that the dosing given is inadequate for the treatment of malaria in the African population. This could even be considered as a "build-in counterfeited drug". The results of these investigations were presented at the 2006 annual meeting of the American Society of Tropical Medicine & Hygiene [7]. Warnings about these findings were also sent to WHO officials and to Medicines for Malaria Venture (MMV).

After the forced withdrawal in 2006 of artemisinin-based monotherapies for malaria under impulse of WHO, the story of DHA and of Cotecxin could have stopped. However, the product was replaced on the market by a new product DHA-Piperaquine in a fixed dose combination tablet under the brand name Duo-cotecxin. This ACT formulation was strongly supported by MMV under a public-private partnership, in which MMV invested many millions of dollars. In order to know whether this new product would be fulfilling the basic pharmaceutical requirements, a sample bought at pharmacies in Kenya was analysed and showed again unsatisfactory results. In fact, when subjected to stability testing (30°C/75% relative humidity), values of 88.6% at t = 0 months and 85.7% at t = 3 months were already below the minimum quantity required, and the typical side and breakdown products of DHA could be found. A few years later, fresh samples were purchased from Kenyan pharmacists and sent to SGS laboratories in Belgium. This laboratory is internationally recognized as a top class centre for quality and its results could not be disputed. For the first batch tested, the active pharmaceutical ingredient (API) content was only 84% and dropped to 73% at the end of shelf-life. The second batch tested, with 99.2% API content, looked rather reassuring. However, when the tablets were subjected to standard accelerated stability testing (40°C and 75% relative humidity), which is a prerequisite for any tablet formulation and results must comply for regulatory purposes, already after one month the content API had dropped to 88.6%. This is below the criterion of 90% of API at the end of shelf life (Table 2).

**Table 2:** Outcome of assay of DHA content in two batches of commercial DHA-Piperaquine tablets (Duo-cotecxin) using method A (HPLC) as described in the International Pharmacopoeia (4^th ^edition)

Batch	Assay (90%-110%)	Status	Storage conditions	Assay (90%-110%)	Status
171007	84.0% *(Dec '08)*	Fail	Room temperature (20°C)	73.0% *(Oct '09)*	Fail
611008	99.2% *(T = 0)*	Pass	40°C and 75% RH (accelerated stability)	88.6% *(T = 1 month)*	Fail

The date of analysis is given between brackets.

Since this process was going on over the last few years, WHO officials were informed about the instability of DHA. They acknowledged the information and confirmed that a series of experts were working on these questions (L. Rägo, personal communication). But surprisingly, another joint venture was subsequently started between MMV and the Italian company Sigma Tau to work on the same combination. MMV was questioned about this discrepancy and they argued that if one started with appropriate Good Manufacturing Practice (GMP) DHA and respected GMP rules during pharmaceutical manufacturing, the breakdown of DHA would not occur (C. Hentschel, personal communication). Unfortunately, the argument about the intrinsic chemical instability of DHA did not score at the time. Requests for receiving a sample of this high quality GMP DHA tablets were refused at several occasions.

Solving the problem of pharmaceutical stability of DHA would be of great importance in view of the role these pharmaceutical formulations may have. However, any underdosing is morbid and pharmaceutical legislation has an important role to play to safeguard the patients. There are few examples in modern pharmaceutical history that demonstrate this point more clearly.

As it stands today, the weight of evidence suggests that none of the available pharmaceutical ACT preparations containing DHA fulfil criteria. It is remarkable that leading journals, such as The Lancet, gave such large volume of printing space to clinical results obtained with an inadequate pharmaceutical preparation [8-10]. Worse, how is it possible that WHO in its recommendation handbook for malaria treatment publishes and recommends an inadequate drug?
